# Too Many Doctors; Efforts to Elevate the Standard of Medical Education

**Published:** 1882-05

**Authors:** 


### TOO MANY DOCTORS. EFFORTS TO ELEVATE THE STANDARD OF MEDICAL EDUCATION.

A physician of Brooklyn was arrested recently for picking a lady's pocket in a street-car. He was found to be one of the numerous poor devils with M.D. attached to their names, who, to save their lives, cannot drum up a practice. Dr. Lamson, the English-American murderer, was driven to crime by his necessities. He could not earn his salt in the practice of medicine. There are hundreds of M.D's. in New York and Brooklyn who are almost starving to death. Many of them do not deserve to succeed. Many of the charlatans, quacks and incompetents fare badly, while others flourish finely right alongside well equipped and worthy professionals who can hardly keep soul and body together. Two facts have for years been painfully apparent to the profession in New York. The ranks are fearfully overcrowded, and the standard of competency is altogether too low.

The profession is now trying to remedy the latter evil, expecting that if the standard of medical education be elevated the evil of excessive numbers will be abated. Bills now before the New York legislature provide that the future graduates of all medical colleges, although holders of their several diplomas, shall be required to stand an approved examination subsequently, before one of several boards of examiners to be appointed by the regents of the University of New York. They prohibit persons from practicing medicine unless their diplomas issued by their medical colleges are reinforced by such a second diploma. They include other measures for elevating the standard of medical proficiency.

On Thirteenth street, between Second and Third avenues, just one block, I recently counted the signs of fifteen physicians. There is no trade or profession in New York that is more overcrowded than the ranks of the medical men. The doctors and surgeons who are making fortunes may be counted on one's fingers. Perhaps one in ten of the profession are earning first-rate livings. The great majority find their professional returns meager enough. Many of them could not live on the scanty doctor's fees they receive. They combine some other business with doctoring, or engage in some branch of the medical art outside the limits of legitimate practice. Not a few doctors' wives support the family by keeping boarders. I could mention the names of two or three men of fair medical attainments, who have so utterly failed to build up a desirable practice that they have moved into the neighborhood of cheap bawdy-houses for the purpose of securing the lowest sort of practice. There are a good many men, not recognized by the regular profession, who were originally ambitious to build up a legitimate practice, but whose adversities have so crushed and debased them that they have become charlatans, quacks and abortionists, not so much by choice as from a grinding necessity to win bread by foul, if they cannot by fair means. Then in the ranks there are hundreds whose general attainments are meager, and whose special medical training is no better. They are humbugs by nature and practice, and able to impose only on the weakest and most illiterate of the people.

It is true that "there is room enough higher up," but this is very poor encouragement for ninety-nine in a hundred medical students, for do what they will, in New York city at least, they never can get "up higher." The men who achieve great success there in medicine are men of marked individuality, who have qualities of mind and manner which raise them inevitably above the mass. They would have succeeded just as well in whatever other pursuit they had chosen to enlist their energies. Many of them had a terribly bitter struggle at first. One physician whose income is $60,000 per annum earned an average of less than $700 a year for the first three years of his practice. I think that his great success is due more to his knowledge of human nature and his splendid business ability than to his proficiency in medicine. But the majority of young medical graduates who go there full of hope and enthusiasm, totally ignorant of the fact that medicine is already overcrowded, confidently expecting a brilliant career, find the bitterest difficulties in the way of their progress. There are a good many of them who, after a few years' conflict with inexorably adverse circumstances, abandon the fight defeated, and return to industries which will at least put bread into their mouths.

The prominent physicians think that the establishment of examining boards, having a tendency to lift the standard of medical culture, will prevent many of these sad life histories, by increasing the difficulty of getting into the profession, and thus removing one of the temptations to the study of medicine by young men without preparatory training.

